# The Balanced View of the Value of Conscience

**DOI:** 10.1111/japp.12655

**Published:** 2023-03-29

**Authors:** Doug McConnell, Julian Savulescu

**Affiliations:** ^1^ Macquarie University North Ryde NSW Australia; ^2^ University of Oxford Oxford UK; ^3^ National University of Singapore Singapore

## Abstract

On the mainstream view, consciences are valuable because they promote moral unity. However, conscience, so defined, will systematically prevent moral growth that threatens unity, even when unity has formed around oppressive moral values. This motivates Carolyn McLeod's alternative ‘Dynamic View’ whereby consciences are valuable to the extent that they are dynamic. Consciences are dynamic when they interact with our best moral judgements to shape or ‘retool’ the moral values underpinning conscience, sometimes at an initial cost to unity. We modify and extend McLeod's account in two ways: (1) We object to her claim that conscience encourages its own retooling. We argue that the opposite is true – conscience creates a motivational barrier to change that moral judgement must overcome to successfully retool conscience. The task of ensuring dynamism, therefore, falls to moral judgement. (2) However, this motivational barrier enables conscience to play a valuable role that McLeod overlooks – compensating for the limitations of moral judgement. On our Balanced View, the value of conscience depends on it being sufficiently open to being shaped by our best moral judgements but inert enough to compensate for distorted moral judgements and to guide action when under cognitive load.

## 1 Introduction

There is a long‐running dispute over the value of conscience with important implications for how to weigh the protection of conscience against other values, such as public health. The dominant account is the ‘Unity View’ which holds that conscience functions to promote moral unity and, because moral unity is necessarily valuable, all consciences are valuable.^1^ However, the Unity View faces significant objections. The relationship between conscience and moral unity is questionable because acting on conscience sometimes undermines unity and, at other times, unity is maintained despite acting against conscience.^2^ In any case, moral unity itself is not necessarily valuable because it can form around substantively immoral values.^3^ Furthermore, a conscience that functions to maintain moral unity will prevent moral growth when new moral knowledge threatens that unity.^4^

Carolyn McLeod's ‘Dynamic View’ is a promising alternative to the Unity View.^5^ She argues that conscience functions to promote the moral values we have internalised (sometimes at a cost to unity) but the value of conscience depends on its dynamism. Conscience is dynamic when it is in a two‐way interaction with moral judgement. Moral judgement adjusts the internalised moral values underpinning conscience to enable moral growth, while conscience contributes to that process by alerting us to the internalised moral values requiring adjustment. A dynamic conscience is, therefore, less likely to get ‘stuck’ promoting moral values that we happen to have internalised but no longer endorse.

We agree with McLeod that the function of conscience is to promote our internalised moral values (not moral unity) and that the value of conscience depends on its interaction with moral judgement; however, we characterise this interaction differently. We begin by arguing, contra McLeod, that conscience does not encourage reconsideration of our internalised moral values but creates motivational barriers to such reconsideration; it thereby works against its own dynamism. At first glance, this inertia appears to undermine the value of conscience since it no longer supports moral judgement in motivating changes to one's internalised moral values. We show, however, that this inertia is an unavoidable aspect of the valuable role conscience plays in guiding action when moral judgement is compromised. McLeod overlooks this valuable role because she focuses exclusively on how conscience should react to our *best* moral judgements. If our moral judgement was always at its best, then we would want conscience to be maximally dynamic in response to those judgements but, unfortunately, we often make sub‐optimal moral judgements. On our ‘Balanced View’, the value of conscience depends on its dynamism being correctly balanced – sufficiently dynamic to change in light of our best moral judgements but not so dynamic that it cannot compensate for compromised moral judgement.^6^

## 2 Common Ground

Before we discuss the function and value of conscience in detail, it will help to set out the features of conscience that most contemporary accounts agree on. Conscience is ‘a capacity … to sense or immediately discern that what [one] has done, is doing, or is about to do (or not do) is wrong’.^7^ Conscience does not, however, indicate which actions are *objectively* wrong, but which actions are wrong by our own lights. That is to say, conscience is subjective and pluralist; it reflects whatever moral values the bearer has committed to and only alerts us to the wrongfulness of our own actions, not those of others.^8^ Conscience motivates action in accord with our moral commitments by threatening us with guilt and shame if we violate, or consider violating, those commitments;^9^ that is, it ‘encourages us to do what we think we morally ought to do by actively discouraging us from the opposite’.^10^ In doing so, conscience provides an immediate, intuitive sense of whether our actions are wrong. This ‘voice’ of conscience does not readily respond to executive control but typically comes unbidden and will ‘nag’ us if we try to ignore it. Despite its intuitive aspect, conscience depends on moral judgement. Taken alone, conscience is a bare commitment to morality, so we rely on moral judgement to fill in the detail of what morality demands.^11^ Moral judgements about the objective content of morality themselves range from the intuitive to the highly reflective, and perhaps even reflective moral judgements are ultimately grounded by moral intuitions.^12^ Intuitive moral judgements about the content of morality – for example, incest is wrong – are different in kind to the intuitions generated by conscience because the latter alert us to our moral commitments, whatever their content. The necessary involvement of moral judgement in the development of conscience does not, however, guarantee that we develop critically informed consciences because we may adopt others’ moral judgements uncritically and our own moral judgements may be flawed. In contrast to conscience, moral judgement is under greater executive control, can assess the morality of others’ actions as well as one's own, and is not limited to detecting blameworthy actions but can assess the moral valence of any action. Given the role of prior moral judgements in the development of conscience, the recommendations of conscience and concurrent moral judgements typically align. When the voice of conscience warns us against an action, upon reflection, we usually endorse those warnings. But how should we understand and resolve situations where the recommendations of conscience clash with the conclusions of our reflective moral judgements? The Unity View, the Dynamic View, and our own Balanced View each offer different answers.^13^

## 3 The Unity View and Its Problems

The Unity View holds that conscience functions to promote ‘unity’,^14^ ‘integrity’,^15^ ‘wholeness’,^16^ or ‘personal integration’.^17^ According to the Unity View, conscience is valuable because it promotes moral unity. The explanatory benefits of the Unity View are: (1) It fits with conscience exclusively alerting us to blameworthy actions because such actions threaten unity while permissible or praiseworthy actions do not threaten unity. (2) It explains that pangs of conscience come unbidden and cannot be easily suppressed because unity is a necessary condition for moral agency; like breathing, we do not usually attend to it unless it is threatened but, when it is threatened, we must prioritise it before we can attend to anything else. (3) It can make sense of claims the folk make, such as, ‘I would not be able to live with myself if I did that’, since serious violation of unity could be such that the guilt, shame, and loss of self‐respect are debilitating. (4) It explains why conscience sanctions one's own behaviour and not that of others because others’ actions do not affect one's own moral unity.

Nevertheless, the Unity View of the function and value of conscience faces problems. The function of conscience as unity‐promoter is suspect because the recommendations of conscience and moral unity come apart. We can maintain moral unity despite acting against conscience by rationalising, finding excuses, and deflecting responsibility onto others; this enables us to falsely conclude that an action was compatible with conscience.^18^ Furthermore, unity can be lost *because* of acting on conscience. McLeod illustrates this with the case of a whistle‐blower who, acting on conscience, complains to management about the sexist culture of her work.^19^ Unfortunately, management does nothing, her colleagues find out about her complaint and harass her even more, while her female colleagues are too afraid to support her. In the end, she is unable ‘to persist in believing that … what she has done is morally right [and ends up] full of self‐loathing and regret, with less moral unity than she started out with’.^20^ So, although acting on conscience promotes moral unity much of the time, unity can be maintained despite not acting on conscience, and acting on conscience does not necessarily promote unity. If conscience only coincidentally or imperfectly promotes moral unity, then the value of conscience *qua* unity‐promoter is undermined.

In any case, there are also reasons to doubt the value of moral unity. Moral unity can form around substantively immoral positions, such as those based on racism or sexism. Furthermore, by promoting moral unity, conscience prevents moral growth that would disrupt unity. Consider a housewife whose conscience reflects the gender norms she grew up with and threatens her with guilt and shame when she considers not cooking dinner for her husband.^21^ Later in life, she learns of more progressive values whereby wives are not obliged to cook for their husbands and, upon critical reflection, judges these to be morally right. However, to adopt those progressive values, she would have to violate her conscience because they undermine established moral unity. The potential for moral growth is valuable, not just because we might discover arguments that show our existing moral beliefs to be false, but because changes in life circumstances can demand significant reprioritisation of the moral values we have internalised. When becoming a parent, for example, the new obligation to care for one's child may supplant obligations that had previously been central to one's life. The value of conscience is significantly undermined if it systematically prevents moral growth.

Perhaps proponents of the Unity View can come up with defences to these objections. Our goal here is not to land a knock‐out blow against the Unity View but to motivate what we take to be the more promising direction taken by McLeod. McLeod offers an alternative view of the function and value of conscience that can accommodate the whistle‐blower and housewife cases.

## 4 McLeod's Dynamic View

McLeod argues that conscience functions ‘not to preserve inner unity, but to encourage us simply to act on our [internalised] moral values’.^22^ It does this in the familiar way, by generating feelings of guilt and shame if we act, or consider acting, contrary to the moral values we have internalised. This view of the function of conscience can easily accommodate the whistle‐blower case because it abandons the idea that conscience necessarily promotes moral unity; by encouraging us to act on our moral values conscience can undermine or even destroy moral unity. But what about the housewife case? If we have internalised moral values that we no longer endorse, a conscience that simply promotes those values would prevent moral growth in the same way as a conscience that promotes unity. McLeod addresses this concern by arguing that the value of conscience comes apart from its function.

For McLeod, conscience is valuable to the extent that it encourages us to act on moral values that we (would) endorse with our best moral judgements.^23^ McLeod defines our best moral judgements as those that are free from ‘psychological oppression’, are grounded in ‘good reasons’ that can be understood by others, and add to ‘the social debate about the nature of right and wrong’.^24^ For ease of exposition, we will call the hierarchy of moral values that people (would) endorse with their best moral judgement their ‘*authentic* moral values’ and those that they would not endorse with their best moral judgement ‘*inauthentic* moral values’.

Conscience encourages us to act on our authentic moral values in two ways. First, to the extent that we have internalised authentic moral values, conscience will motivate us to act in accord with them. If this was all that conscience could provide, its value would be contingent on whether we happen to have internalised authentic values. However, conscience contributes to the internalisation of authentic values through its second feature: ‘Even when our conscience is a voice of oppression (or abuse or the like), it can have a positive effect, that is, by alerting us to having some oppressive or otherwise bad values that may be unconsciously influencing our behaviour’.^25^ Without conscience to alert us to oppressive values we have internalised, their unconscious influence on our behaviour would go unchecked.

To illustrate, my conscience could encourage me to prepare dinner for my husband most nights, simply because he is my husband. It would do that by threatening me with false guilt or shame … that do not reflect what I actively endorse … *My conscience would allow me to see how much these values are part of me and could prompt me to reflect on how I was relating to my spouse because of them*. Say that I'd been making his dinner repeatedly, but then wanted a break from it. Taking a break is not as easy as I thought it would be, because my conscience starts to nag me about preparing dinner. I say to myself, ‘But why should I feel guilty about not making dinner? … I should reject these attitudes and resist getting into patterns of “wifely” behavior, rather than allow them to develop’.^26^

By alerting us to otherwise unconscious, oppressive values, conscience ‘jump‐starts the process of retooling’ conscience,^27^ that is, the process of increasing the internalisation of authentic moral values and externalising inauthentic moral values. McLeod does not specify exactly what she means by ‘internalisation’ but, presumably, it refers to how deeply a moral value is embedded in one's sense of self, such as how thoroughly it is integrated with one's other self‐beliefs or self‐narrative. Retooling is directed by reflective moral judgement and can take months or years to complete when inauthentic moral values are deeply internalised. ‘A conscience is *dynamic* in the … sense [that] it will change when it conflicts with what we judge to be morally correct’.^28^ So, although conscience will motivate pursuit of whatever moral values one has internalised, it is valuable when it contributes to a reflective process through which the agent can increase the internalisation of authentic moral values. The housewife's conscience has value to the extent that it prompts reflection on her internalised moral values and is open to being retooled by her best moral judgement.

Although a dynamic conscience has the valuable potential for moral growth that an inert conscience lacks, McLeod recognises that dynamism alone will not ensure that one's conscience comes to reflect one's authentic moral values. Some people's moral judgement might shape conscience to become (even more) oppressive, especially if they are immersed in a ‘noxious social environment’.^29^ For McLeod, such consciences lack almost all value despite being dynamic;^30^ consciences are only valuable when they are dynamic in response to our *best* moral judgements, that is, judgements grounded in good reasons that others can understand.

A key difference between the Unity and Dynamic views, then, is in how they characterise the interaction between conscience and moral judgement. On the Unity View, although moral judgement is involved in choosing which moral values to commit to,^31^ once we have committed, moral judgement and conscience have different remits and do not interact. Conscience promotes moral unity by preventing us from acting against deep moral commitments, while moral judgement helps us choose between actions that are compatible with our moral commitments. In cases where conscience makes two conflicting recommendations of similar strengths, that is, when we face a choice between two evils, then moral judgement can settle which should be given priority.^32^ Proponents of the Unity View do not tend to consider cases where conscience and moral judgement come into conflict. Sulmasy even seems to preclude the possibility when he says, ‘if conscience is derived from the fundamental moral commitment to choose the right and the good, then one is obligated to choose what one has determined to be the right and the good’.^33^ This seems to ignore the possibility that we might also determine what is right and good with a moral judgement that could clash with conscience. In any case, the Unity View implies that moral judgement should fall in line with conscience because moral unity is so important. On the Dynamic View, in contrast, ‘conscience and moral judgement can and should influence one another’ throughout our lives.^34^ Indeed, the value of conscience is conditional on us reflectively retooling it when we encounter new moral knowledge; without the involvement of moral judgement, conscience would only coincidentally promote our authentic moral values.

We agree that the value of conscience depends on its ongoing interaction with moral judgement but we characterise that interaction differently. In the following section, we begin by arguing that conscience only plays a passive role in alerting moral judgement to internalised moral values and, rather than promote retooling, it creates motivational barriers to changing our internalised moral values. Therefore, conscience contributes significantly less to its own dynamism than McLeod attributes to it.

## 5 Deflating the Role of Conscience in Its Own Dynamism

McLeod claims that ‘conscience has value … when it *forces us to reconsider and revise* some of [our moral] values’.^35^ Conscience contributes to its own retooling by ‘prompting’ or ‘alerting’ us to ‘oppressive or otherwise bad values that may be unconsciously influencing our behaviour’ and this ‘jump‐starts’ reflection on those values.^36^ When the wife feels guilt and shame at the prospect of not cooking her husband dinner, for example, she is reminded that she has internalised moral values that she does not endorse and is motivated anew to change them.

We think that this overstates the role of conscience in the retooling process. Conscience does not motivate the reflective retooling process, but rather discourages us from drawing reflective conclusions contrary to our internalised moral values. This damper on reflective retooling is part of how conscience discourages actions contrary to our internalised moral values, and thereby fulfils the function that McLeod (and we) attribute to it. Conscience does not usually wait until such actions are imminent (or have occurred) to generate guilt and shame, but begins to do so when we take cognitive steps towards such actions, for example, by considering or committing to intentions, policies, or conceptions of the good life that motivate and make sense of. Indeed, given the intimate connection between action and cognition, this is what we would expect from a conscience effectively fulfilling its function. The internalised moral values making the wife feel guilt and shame about not cooking dinner, for example, would also threaten her with guilt and shame if she seriously considered committing to the view that wives are not obliged to cook for their husbands.^37^ Therefore, to the extent that conscience elicits guilt and shame when we consider committing to new moral values, it discourages moral judgement from retooling our oppressive moral values.

Furthermore, the role conscience plays in motivating us to think and act in accord with our internalised moral values will tend to pre‐empt self‐reflection. Critical reflection is effortful and we prefer to do no more than necessary,^38^ so we tend to think and act in accord with our intuitions and established values, plans, intentions, beliefs, etc., unless something interrupts or challenges our patterns of action, or we are uncertain of how to proceed. Therefore, the threat of guilt and shame only moves us to reflect on why we feel those emotions when the conflict between underlying values and our (potential) actions is sufficiently large or frequent. This is what we see in McLeod's example; the wife is already largely committed to a lifestyle that does not prioritise cooking dinner for her husband and *in that context* her feelings of guilt and shame for not cooking dinner strike her as particularly alien. She is also confident that her judgement that wives are not obliged to cook dinner for their husbands is her best moral judgement; she has reflected upon it extensively, found a number of reasons for thinking it to be true, and defused possible objections. In stark conflicts like these, a person can readily question the recommendations of their conscience with the persistence required to conclude that the underpinning moral values are false. However, relatively small or infrequent conflicts between the internalised moral values underpinning conscience and action may be insufficient to motivate sustained reflection, so the agent might prematurely and incorrectly conclude that the moral values underpinning her conscience are authentic, especially if she lacks confidence in her moral judgement on the issue at hand. In such cases, it will seem unlikely to her that this could be her *best* moral judgement given the shame and guilt it generates, so she will tend to take the voice of conscience at face value and the ‘jump‐starting’ of the reflective process required for retooling will not occur.

Therefore, verbs like ‘force’, ‘jump‐start’, or ‘prompt’ for how conscience influences reflection on and retooling of internalised moral values are misleading because they suggest a sense in which conscience *motivates* reflection and retooling. The role of conscience in its own dynamism is more passive than that. Although conscience generates guilt and shame unbidden, this only leads to reflection on one's underlying moral values when one considers actions that conflict sufficiently seriously with internalised moral values. Furthermore, for that reflection to be sufficiently persistent to lead to the conclusion that those internalised values should be retooled, then one must be certain enough in one's moral judgement to motivate reflection *despite* the recommendations of conscience.

Nevertheless, we accept that there remains a way in which conscience *enables* reflection on the moral values generating those emotions *relative to* cases where internalised moral values do not generate guilt and shame at all. In the latter cases, it would be especially difficult to retool one's inauthentic moral values because it would be more difficult to become aware of their influence and, without being aware of them, one could not bring one's executive capacities to bear on them. In other words, having a system that alerts us to conflicts between our internalised moral values and our (potential) actions, even as it discourages us from changing them, is better than not having such a system at all.

A couple of variations on McLeod's example can help us appreciate why this passive feature of conscience is still of some value. When we think of how conscience might alert us to internalised moral values exerting unconscious influence, there are degrees of unconsciousness. Moral values are more deeply unconscious when the bearer would struggle to identify them correctly upon reflection. Moral values are unconscious in a shallower sense when the bearer could readily identify them but is not attending to them right now. In the example that McLeod uses to illustrate her view, the internalised moral values are unconscious in a shallower sense. The woman who feels guilt and shame for not cooking her husband dinner can readily identify the source of those feelings, namely, the internalised gendered expectations of how wives should care for husbands. The experience of guilt and shame when she considers not cooking reminds her that she still has those values and (contra the motivations of conscience) will need to continue to externalise them. But we think that conscience can also help us articulate moral values that are unconscious in the deeper sense. Conscience might generate shame and guilt which, upon reflection, we cannot clearly attribute to any of our familiar moral values. Unexplained shame and guilt tend to be surprising and confusing, so these cases will tend to trigger a reflective search for the kind of moral value that could explain how we feel. A person may not realise, for example, that she has internalised a set of gendered expectations of how wives should care for husbands. When she reflects on the contexts in which she feels this unexplained guilt and shame, such as when she considers not cooking for her husband, she may then realise that she has internalised these moral values after all.

Conscience can also help us develop a more detailed understanding of *authentic* moral values we have internalised. This is most obviously the case with deeply unconscious authentic moral values because it is not until unexplained guilt and shame have motivated us to identify them that we can begin to explicitly pursue them. Even when we are familiar with our authentic moral values, the strength with which shame and guilt are elicited by considering going against those values might surprise us, leading us to recognise the true magnitude of their value to us.

So, we accept that conscience contributes to the interaction with moral judgement by sometimes alerting us to conflicts between our (potential) actions and the moral values we have internalised. This passive, reflection‐enabling effect of conscience is of some, albeit lesser value than it would be if it could motivate persistent reflection on inauthentic values. Perhaps this is what McLeod meant all along, but it is important to recognise that any reflection enabled by conscience in this way does not provide motivation to change conscience. In fact, conscience works against its own dynamism by encouraging us to follow our internalised values until conflicts arise and by recommending against changing those values in cases where we do reflect on them. In other words, conscience creates motivational barriers to its own change so whatever dynamism conscience has must be driven by moral judgement.^39^

This appears to make the value and dynamism of conscience almost entirely parasitic on moral judgement, since only moral judgement can distinguish authentic from inauthentic moral values and drive the internalisation of the former and externalisation of the latter. However, in the next section we show that the inertia of conscience provides a valuable role that moral judgement cannot – it compensates for weaknesses of moral judgement.

## 6 Conscience Can Compensate for Compromised Moral Judgement

McLeod focuses on cases where conscience reflects oppressive moral values to show how our best moral judgement can helpfully shape conscience. We agree that this process is important, but by focusing exclusively on these cases, conscience is made to appear excessively subordinate to moral judgement. In this section, we point out that our moral judgement is often compromised. It may be unavailable due to cognitive load or distorted by a range of factors. Therefore, the relatively automatic and stable influence of conscience, that is, the inertia of conscience, sometimes promotes our authentic moral values better than moral judgement.

The first kind of case where conscience has the potential to be better than moral judgement at guiding moral behaviour is where the agent is under heavy cognitive load. Conscience does not just motivate actions that align with our internalised moral values; it does so with greater cognitive efficiency than making reflective moral judgements. To act on conscience, we simply need to take the shame and guilt it generates at face value and avoid actions that generate those emotions. When we do so, we assume that these emotions align with what we would judge to be blameworthy if we deliberated. We can be guided by conscience in this way even when we are not sure which of our internalised moral values is guiding our action. Moral judgement typically requires much more in the way of cognitive resources to work out which of several possible actions is best or whether the current course of action is justified. This involves, *inter alia*, imagining how one or more actions would turn out, including others’ reactions, and assessing trade‐offs between moral values, such as how to balance honesty and benevolence or when to support a family member's interests over those of a friend or a stranger.

When not under cognitive load, it might be negligent to follow one's conscience in a completely unreflective way. But we are often under cognitive load when morally challenging situations arise. If there are not sufficient resources to make a moral judgement, then having conscience guide action in accord with one's internalised moral values is significantly better than nothing. If we could not rely on conscience, presumably we would make many more moral errors as the demands of morally charged situations outstripped available cognitive resources.

By letting conscience be our guide, more cognitive resources are available for other tasks. In some cases, these extra cognitive resources might be necessary to meet moral norms. They might, for example, enable one to recognise the morally relevant features of a situation. If one does not recognise what is at stake morally, then one risks damaging others’ interests through culpable ignorance. In other cases, the extra cognitive resources might enable us to achieve valuable self‐interested goals that require our full attention. If we had to make reflective moral judgements while pursuing such goals, the cost in cognitive resources would cause us to fail or perform sub‐optimally. The lower cognitive demands of conscience allow us to maintain moral standards while successfully pursuing such challenging goals.

The second kind of case where conscience is better than moral judgement at guiding moral behaviour is where our moral judgements are sub‐optimal. Sometimes we let ourselves be too easily convinced of a moral belief; perhaps we are swayed by the charm and good looks of a charismatic interlocutor so that we fail to properly attend to the quality of the relevant arguments and evidence. At other times, our desire to fit in socially might lead us to adopt the group's moral views without properly reflecting on them. Similarly, we might commit too readily to unjustified moral beliefs, perhaps because they are more compatible with our self‐interested goals.

There is also suggestive evidence for a phenomenon called ‘judgement shift’, where our judgement is warped through exposure to temptation.^40^ Temptations are options that we find desirable but judge less valuable than other mutually incompatible options; for example, we have to resist late‐night drinking with friends in order to go skiing tomorrow morning. In judgement shift, one's judgement shifts in favour of the tempting option, sometimes to the point that it is no longer seen as a temptation but just as the all‐things‐considered best option. One later regrets that choice once judgement returns to its pre‐temptation baseline. Since a subset of temptations conflict with our moral commitments, presumably they will shift moral judgement. For example, one might have to resist late‐night drinking with friends in order to fulfil one's promise to look after one's children tomorrow morning. If judgement shift takes hold, then one's moral judgement might temporarily change to conclude that, in this case, it is morally permitted to break one's promise.

Conscience can protect us from sub‐optimal moral judgements, whatever their cause, by threatening guilt and shame when we consider courses of action that go against our internalised values. The threat of guilt and shame can guide action before a person deliberates, or it can prompt further deliberation which leads to a better quality moral judgement. To illustrate, consider the case of a lonely young man who starts interacting with an online group. He initially enjoys the social interaction but soon it becomes clear that this group are committed white supremacists. When the group invite him to an in‐person social event, his conscience threatens sufficient guilt and shame at the prospect of socialising with racists that he declines without serious deliberation, so his reflective moral judgement is not even engaged. However, we can imagine that he is so desperate for a wider social network that, *if he had deliberated*, judgement shift would have led him to conclude that attending the event was morally permissible after all. In this case, his conscience has ensured that he acts on his internalised authentic moral values when his moral judgement would not have.

Now, consider a second lonely young man who has not internalised anti‐racist values so deeply. He is lured in by the white supremacist group and begins to attend their in‐person events where he finds acceptance, camaraderie, and a feeling of power. He is exposed to a range of arguments for the view that multiculturalism is the cause of many of society's ills. Never having thought much about racial purity or multiculturalism, he takes these arguments at face value and, in any case, he wants to fit in with the group. Some of the group's activities promoting racial purity, such as putting racist posters in public spaces, pique his conscience but he readily dismisses these feelings. One day, however, he agrees to accompany his friends to threaten a local shop owner from a minority ethnic background. Before entering the shop, he judges that the ends justify the means, but once he sees the frightened reaction of the shopkeeper his conscience elicits such strong guilt and shame that he cannot join in. This leads him to question his new anti‐multicultural judgements; he now sees that they are incompatible with other values he is committed to, for example, that others should be treated as free and equal. Upon further reflection, he becomes convinced that his conscience is right and he was lured into incorrect moral judgements through his need to feel part of a group.

This second lonely young man's conscience does not protect his internalised authentic moral values as well as that of the first lonely young man. The guilt and shame it initially elicits are too weak to prevent him judging that racial purity is a morally permissible goal but it eventually elicits enough guilt and shame that it motivates persistent critical reflection. This then enables him to detect and correct his sub‐optimal moral judgements. Although moral judgement, not conscience, is ultimately required to work out whether to stand by the moral values underpinning conscience,^41^ it is the influence of conscience that motivates him to put the effort into making a better quality moral judgement.

The influence of conscience can also improve the quality of our moral judgements over time. If conscience fails to override a flawed moral judgement in the moment, the guilt and shame when we realise our error motivate us to take steps to improve the quality of our future moral judgements. The second lonely young man, for example, might realise that his need for social connection impaired his moral judgement. He might then resolve to be alert to similar mistakes in the future and take steps to develop a less noxious social network which would reduce his vulnerability to such errors of judgement.

In sum, the *inertia* of conscience allows it to act as a valuable counterbalance to compromised moral judgement; it can guide action in accord with our internalised authentic moral values when cognitive load makes moral judgement difficult and when sub‐optimal moral judgements would have us act against those moral values. Furthermore, conscience can motivate us to improve the quality of our moral judgements over time. We can note that these valuable aspects of conscience are not entirely parasitic on moral judgement. Although we depend on making our best moral judgements to ensure that conscience reflects authentic moral values rather than inauthentic ones, conscience provides those authentic values with a diachronic stability that moral judgement cannot.

If conscience is valuable because of its inertia, how does this fit with McLeod's view that consciences are valuable when they are dynamic? In the next section, we describe the implications of our analysis for attributing value to conscience and show how our position differs from the Dynamic View.

## 7 The Value of Conscience Depends on Its Balance with Moral Judgement

On the Dynamic View, consciences are valuable when they are dynamic in response to our best moral judgements and they lack value when they are dynamic in response to poor moral judgements; for example, those that stem from ‘psychological oppression’ or that cannot be understood by others. Our analysis is compatible with those claims but we have added that consciences are valuable when they exhibit a degree of inertia because inertia protects us from acting on compromised moral judgements and retooling conscience in light of such judgements.

Our observation that the inertia of conscience is sometimes valuable can be partly incorporated into McLeod's view as it stands. She notes that one valuable aspect of conscience is that it encourages us to act on our authentic moral values to the extent we have internalised them. That motivation is valuable when it backs up our best moral judgements because it tends to ensure we act on those judgements. For example, if I judge that eating meat is wrong, I will be more likely to act on that judgement if it is backed by conscience. But this motivation is *also* valuable when it precludes or overrides compromised moral judgements; for example, by preventing me from eating meat when judgement shift would temporarily cause me to judge that eating meat is permissible.

However, once we see that conscience can preclude or override moral judgements, it follows that, to the extent we have internalised *in*authentic moral values, conscience will not only motivate morally inauthentic behaviour (as McLeod recognises) but it will also tend to preclude or override our best moral judgements, as we have argued in Section 5. So, we face a trade‐off between having a conscience inert enough to compensate for compromised moral judgements but not so inert that it will override well‐justified moral judgements and be impervious to retooling.

We could avoid that trade‐off if we had full understanding of the quality of our moral judgements, as we would then know when to rely on conscience until we could form a better judgement and when to ignore (and work on retooling) conscience. This would enable our consciences to be maximally dynamic in response to our best moral judgements but static in the face of compromised moral judgements. There is some evidence showing that moral judgements come with a metacognitive ‘feeling of rightness’ so that we feel more confident in the ‘rightness’ of those moral judgements that come to us ‘fluently’ than those where countervailing considerations create doubt in the judgement.^42^ Unfortunately, however, we are not perfect metacognitive judges of the quality of our moral judgements. Although we calibrate confidence in our moral judgements so that it is proportionate to the reasons supporting those judgements, we still suffer from underconfidence in relatively well‐justified judgements and overconfidence in relatively unjustified judgements.^43^

We suggest that an analogous uncertainty arises in regard to the voice of conscience. We aim to internalise moral values so that the guilt and shame elicited by going against them are proportionate to the importance those values have for us. However, we do not always get this right, either over‐ or under‐internalising moral values so that the guilt and shame generated are disproportionate to the importance those values have for us. Furthermore, as discussed in Section 5, we are not always aware of the degree to which we have internalised moral values. Knowing that the voice of conscience can threaten excessive guilt and shame in some cases and insufficient guilt and shame in others means that we should take the strength of its recommendations with a grain of salt.

Given the degree of uncertainty attached to both our moral judgements and the voice of conscience, we inevitably face uncertainty in resolving some of the conflicts between them. To illustrate, consider how the conflict between the housewife's conscience and moral judgement developed over time. When she was younger, her judgement that wives should not have to cook for their husbands was easily outweighed by her conscience. She did not have much confidence in that judgement, partly because she was still uncertain about the reasons supporting it and partly because it was difficult to think such a judgement could be correct given the strong guilt and shame it elicited from conscience. But, as she found more reasons supporting her judgements about the role of wives, her confidence in those judgements grew, and she eventually became uncertain as to whether her judgement or conscience was right. At this inflexion point, she sometimes began to act on those moral judgements despite her conscience while, at other times, acting on conscience despite her moral judgements. As confidence in her moral judgement increased further, however, she began to act on moral judgement more consistently and started to retool her conscience so the guilt and shame it would elicit decreased. We can imagine the housewife wishing that her conscience had been more dynamic. If she had placed more confidence in her moral judgements relative to her conscience then she would have reached the inflexion point and moved through it more quickly, but at least her conscience was dynamic enough that she ultimately internalised her authentic moral values on this matter.

Conversely, the second lonely young man was initially overconfident in his moral judgement that his involvement with a racist group was compatible with his authentic moral values and ignored the voice of conscience. Eventually his conscience elicited sufficiently strong shame and guilt that he revised his inaccurate moral judgements, but we can imagine him wishing that his conscience had overridden his flawed moral judgements sooner, that is, exhibited more inertia. Nevertheless, he should be pleased that his conscience was not more dynamic since he would have continued to act against his authentic moral values and might even have eventually started to retool his conscience based on his compromised moral judgements.

Given the risks of having a conscience that is either overly dynamic or overly inert, we should want a balance between the confidence we place in moral judgement and in the voice of conscience. The optimal balance between conscience and moral judgement is one that maximises pursuit of our authentic moral values. On the Balanced View, a conscience is of greater value the closer it is to the optimal balance with moral judgement; consciences are of lesser value to the extent they are overly dynamic or overly inert.

The optimal balance is a function of our environments and our capacities of moral judgement, so it will vary by person and within‐person both over time and by context. In cases where one's moral judgement is rarely distorted, high cognitive load is rare, and new moral knowledge regularly becomes available, then a relatively dynamic conscience would be more valuable because it would allow one to quickly shape conscience in light of new knowledge while not needing to rely on conscience to protect against compromised moral judgements. Conversely, in contexts where one's moral judgement is vulnerable to distortion, high cognitive load is common, and new moral knowledge is rare, a relatively inert conscience would be more valuable because it would consistently protect against compromised moral judgements.^44^ One would have to work harder to achieve moral growth but, as long as the need for moral growth was rare, the trade‐off would be worth it.

To see how someone might benefit from changing the dynamism of their conscience over time, imagine a person who was raised in a cult but escapes and recognises that they have internalised a warped morality that is clearly unsuitable for the world they now find themselves in. They would initially benefit from developing a highly dynamic conscience, placing little weight on the voice of conscience when it clashes with moral judgement. This enables fast retooling but, during this phase, it leaves them less able to compensate for cognitive load and they tend to make moral errors by trusting moral judgement too readily. Later, when their retooling is largely complete, they would benefit from decreasing the dynamism of conscience so that it better compensates for the limitations of judgement. Their capacity for fast moral growth decreases but is no longer needed.

We might also have the capacity to adjust the dynamism of conscience according to more localised contexts. A surgeon might benefit from an inert conscience in the operating theatre where ethical decisions are typically under high cognitive load and lengthy moral deliberation is likely to be detrimental. In this context, the surgeon would only let the most confident moral judgements override the recommendations of conscience. Yet we might hope that that same surgeon, when discussing options prior to surgery or when debriefing after surgery, might have a more dynamic conscience which is more open to being shaped by new moral arguments. Over time, the changes made in reflective contexts would influence conscience‐led behaviour in the theatre.

## 8 Conclusion

We agree with McLeod that conscience functions to promote our internalised moral values, rather than moral unity, and we agree that the value of conscience depends on its interaction with reflective moral judgement. However, we have argued to modify and extend McLeod's account in two ways.

First, although we agree that conscience *enables* reflection on our internalised moral values, we have argued against the claim that conscience motivates its own retooling. When conscience alerts us to a conflict between our actions and our internalised values, it *discourages* reflective conclusions contrary to those internalised values. Conscience thereby creates a motivational barrier to change that must be overcome if moral judgement is to successfully retool conscience. However, this motivational barrier is part and parcel of the value of conscience since, without it, conscience would be too readily retooled by compromised moral judgements and it could not stand in when moral judgement was compromised or unavailable.

Second, we have argued that McLeod overlooks the main value that conscience offers, which is to compensate for the limitations of moral judgement. Conscience can do so by providing a stable moral perspective which complements the more agile, cognitively demanding, and abstract perspective provided by moral judgement. Not only should moral judgement retool conscience where oppressive moral values have been internalised, but conscience should override distorted moral judgements and motivate the development of moral judgement to reduce its vulnerability to being compromised. The value of conscience depends on the bearer achieving a balance between these two processes that best enables pursuit of their authentic values given their capacities and context.

## NOTES

1 Childress, “Appeals to Conscience”; Blustein, “Doing What the Patient Orders”; Pellegrino, “Physician's Conscience”; Benjamin, “Conscience”; Sulmasy, “Conscience.”

2 McLeod, *Conscience in Reproductive Healthcare*.

3 Giubilini, “Objection to Conscience.”

4 McLeod, *Conscience in Reproductive Healthcare*.

5 Ibid.

6 We follow McLeod in restricting the scope of our analysis to the relationship between conscience and moral judgement; however, there may be other aspects of moral psychology that are relevant to the value of conscience. For example, if moral perception can yield moral knowledge, then a valuable conscience should also be dynamic in response to moral perception. Likewise, the voice of conscience might valuably sensitise moral perception. A complete picture of the value of conscience might, therefore, be somewhat more complicated than what we can present here. We thank an anonymous reviewer for bringing this direction for future research to our attention.

7 Hill, “Four Conceptions of Conscience,” 14.

8 Giubilini, “Conscience.”

9 Blustein, “Doing What the Patient Orders,” 297; Hill, “Four Conceptions of Conscience,” 69.

10 McLeod, *Conscience in Reproductive Healthcare*, 34.

11 Lyons, “Conscience”; Sulmasy, “Conscience”; Pellegrino, “Physician's Conscience.”

12 The account of conscience we provide here is compatible with cognitivist and non‐cognitivist foundations of moral judgement.

13 The features of conscience and its relationship with moral judgement described here are not universally agreed upon. Notably, some philosophers have thought that conscience has the power to reveal moral truth. Rousseau (*Emile*), for example, saw conscience as a superior form of moral judgement, while Christian philosophers have seen conscience as a (fallible) means of interpreting the divine law imbued in us by God (Sorabji, *Moral Conscience*). On those views, conscience does not wait on moral judgement but is itself a source of moral judgement, and its value is its power to reveal moral truth. These views have fallen out of favour, however, because it is difficult to ground the epistemic authority of conscience (see Giubilini, *Conscience*, for a more detailed discussion).

14 Benjamin, “Conscience,” 470.

15 Pellegrino, “Physician's Conscience,” 228.

16 Benjamin, “Conscience,” 470; Childress, “Appeals to Conscience,” 322; Blustein, “Doing What the Patient Orders,” 300.

17 Blustein, “Doing What the Patient Orders,” 297; Sulmasy, “Conscience,” 138.

18 McLeod, *Conscience in Reproductive Healthcare*. McLeod also points out that there are at least two plausible versions of the Unity View – one where conscience promotes maximum achievable moral unity and one where it promotes the minimal unity required for moral responsibility (ibid., pp. 27–33). We gloss over this distinction here but McLeod provides detailed objections against both.

19 Ibid., 31–2.

20 Ibid., 32.

21 Ibid., 35.

22 Ibid., 33–4.

23 Ibid., 38–9.

24 Ibid., 39. Although McLeod does not explicitly say so, such judgements appear to meet the standard of Rawlsian reasonableness and have a defeasible claim to tracking moral truth (Boettcher, “What Is Reasonableness?”; Rawls, “Idea of Public Reason”). Racist or sexist moral views, for example, cannot be understood by others or genuinely contribute to the moral debate, so cannot be the outcome of one's best moral judgement so defined. The agent‐centric term ‘best moral judgement’ is, therefore, slightly misleading since some people's best moral judgements might fall short of this standard. For argument's sake, we will assume that everyone has the capacity to make the high‐quality moral judgements McLeod refers to, but those who disagree can read ‘best’ moral judgement as ‘reasonable’ moral judgement.

25 McLeod, *Conscience in Reproductive Healthcare*, 35.

26 Ibid., 35, our emphasis.

27 Ibid., 36.

28 Ibid., 36.

29 Ibid., 39.

30 However, even oppressive consciences retain some minimal value because they represent a commitment to morality and this is better than not being concerned with morality at all (ibid., 39).

31 Pellegrino, “Physician's Conscience,” 227.

32 Childress, “Appeals to Conscience,” 320.

33 Sulmasy, “Conscience,” 140.

34 McLeod, *Conscience in Reproductive Healthcare*, 34.

35 Ibid., 33, our emphasis.

36 Ibid., 35.

37 Indeed, committing herself to such a conception of the good life might generate more guilt and shame than a single failure to cook dinner because it would signal a permanent and more serious break from her internalised moral values. A single instance of not cooking dinner, in contrast, might be more easily rationalised or excused, or the shame and guilt more easily endured. This, however, is an empirical matter that cannot be answered here.

38 Kool *et al*., “Decision Making.”

39 Or other capacities, such as moral perception.

40 Holton, *Willing, Wanting, Waiting*; Dill and Holton, “Addict.”

41 The primacy of reflective moral judgement is not surprising when we recall that we rely on moral judgement to fill in the details of morality for conscience in the first place.

42 Vega *et al*., “Metacognition in Moral Decisions”; Cecchini, “Moral Intuition.”

43 Bago and De Neys, “Intuitive Greater Good”; Miller and Geraci, “Improving Metacognitive Accuracy”; Vega *et al*., “Metacognition in Moral Decisions.”

44 This bears some similarity to the Unity View which one could interpret as favouring a maximally inert conscience. However, even in the rare contexts where maximally inert consciences would be valuable, our view still differs from the Unity View because, like McLeod, we hold that conscience functions to promote our internalised moral values rather than unity.
